# Molecularly diagnosed glioblastoma: clinical presentation and outcomes

**DOI:** 10.1007/s11060-026-05752-8

**Published:** 2026-08-07

**Authors:** Harold F. Hounchonou, Manolis Polemikos, Ariyan Pirayesh, Genis Bajgora, Shadi Al-Afif, Christian Hartmann, Joachim K. Krauss

**Affiliations:** 1 Department of Neurosurgery, Hannover Medical School, Carl Neuberg Straße 1, 30625 Hannover, Germany; 2 Department of Neuropathology, Hannover Medical School, Carl Neuberg Straße 1, 30625 Hannover, Germany

**Keywords:** Molecular glioblastoma, Glioblastoma, High grade glioma, IDH-wildtype glioma, Non-enhancing glioblastoma

## Abstract

**Background:**

The 2021 WHO classification permits diagnosis of glioblastoma in IDH-wildtype diffuse astrocytic tumors with TERT promoter mutation, EGFR amplification, or chromosome 7 gain/10 loss, even without necrosis or microvascular proliferation. The clinical behavior of these molecularly defined glioblastomas (molGBM) remains uncertain.

**Methods:**

We retrospectively analyzed patients with molGBM treated at Hannover Medical School between 2017 and 2024. A contemporaneous histologically defined glioblastoma cohort (hisGBM) served as a control group in an approximately 1:3 ratio. MolGBM were stratified by histological grade and additionally compared with IDH-mutant astrocytomas of corresponding grade. Survival was assessed using Kaplan-Meier analysis, Cox regression, and propensity score matching.

**Results:**

Thirty-four patients with molGBM and 103 with hisGBM were included. Compared with hisGBM, molGBM patients were younger (*p* < 0.001), had higher Karnofsky Performance Status (*p* < 0.001), and more often presented with seizures (*p* = 0.006), whereas focal neurological deficits were more frequent in hisGBM. MolGBM showed less contrast enhancement (*p* < 0.001) and lower Ki-67 indices (*p* < 0.001). Median overall survival was longer in molGBM (539 vs. 374 days; *p* = 0.032), but tumor group was not independently associated with survival after adjustment for age and KPS (HR 1.21, 95% CI 0.77–1.90; *p* = 0.411). In the matched cohort, overall survival did not differ significantly (517 vs. 468 days; *p* = 0.670). MolGBM had inferior survival to grade-corresponding IDH-mutant astrocytomas (*p* < 0.001).

**Conclusions:**

MolGBM shows a distinct clinical-radiological phenotype, while its apparent survival advantage over hisGBM is influenced by baseline clinical differences.

**Clinical trial number:**

Not applicable.

## Introduction

The 5th edition of the World Health Organization (WHO) classification of central nervous system (CNS) tumors incorporated molecular features as key biomarkers for tumor classification and grading [1]. In glioblastoma, WHO CNS grade 4, three molecular alterations - TERT promoter mutation, EGFR gene amplification, and complete gain of chromosome 7 in combination with complete loss of chromosome 10 (+ 7/-10) - were integrated into the diagnostic criteria [1]. According to this definition, IDH-wildtype diffuse astrocytic gliomas that harbor any of these molecular features, and exhibit no histological hallmarks such as necrosis or microvascular proliferation, are now allowed to be become classified as glioblastoma [1]. As a result, histopathological features alone are no longer required to establish the diagnosis. This update was based on recommendations from the Consortium to Inform Molecular and Practical Approaches to CNS Tumor Taxonomy (cIMPACT-NOW) in their third update, which introduced the entity “Diffuse astrocytic glioma, IDH-wildtype, with molecular features of glioblastoma, WHO grade IV” [2]. These recommendations drew largely on studies of lower-grade gliomas that carried such molecular alterations, many of which lacked extensive clinical comparison with conventional glioblastoma [3–8]. Interestingly, some investigators reported longer survival in these tumors compared with conventional glioblastoma, raising the possibility of biological differences between the two groups [4, 8].

Against this background, the present study investigates whether molecularly defined glioblastoma (molGBM) – characterized by TERT promoter mutation, EGFR amplification, or + 7/−10 copy number alterations in the absence of necrosis or microvascular proliferation – differs from histologically defined glioblastoma (hisGBM) with regard to clinical presentation and survival outcomes. We hypothesize that molGBM might represent a less aggressive subtype, associated with a more favorable clinical presentation and improved survival compared to hisGBM.

## Methods

### Study design

We conducted a retrospective review of medical records from all patients diagnosed with glioma at Hannover Medical School between 2017 and 2024. Neuropathology reports were screened to identify patients with tumors corresponding to molGBM, defined as IDH-wildtype diffuse astrocytic gliomas lacking necrosis or microvascular proliferation but harboring molecular features of glioblastoma. Demographic, clinical, radiological, neuropathological, treatment, and outcome data were collected.

**Fig. 1:**
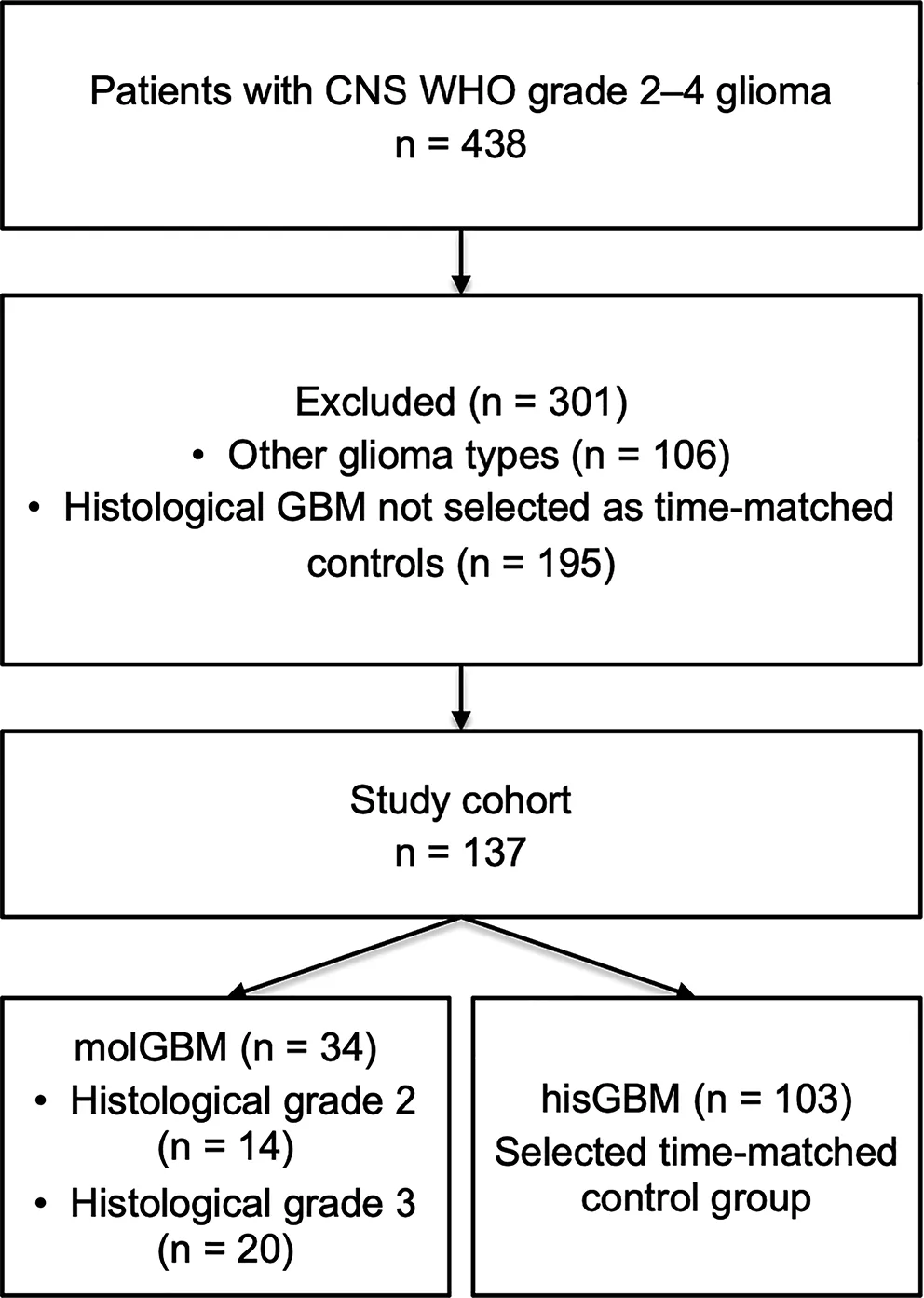
Patient selection flowchart

The primary control group comprised patients with hisGBM diagnosed during the same institutional study period, selected according to the closest date of diagnosis to each molGBM case in an approximately 1:3 ratio. This date-based selection was used to generate a contemporaneous comparison cohort and reduce calendar-time bias related to diagnostic workup, treatment standards, and follow-up duration; controls were not selected based on outcome, treatment response, or baseline prognostic characteristics. Patient selection is summarized in Fig. 1. The primary comparison focused on clinical presentation and OS between molGBM and hisGBM. To further contextualize the prognosis of molGBM, secondary control groups were randomly selected from the institutional database and consisted of patients with IDH-mutant astrocytoma of corresponding histological grade. Specifically, molGBM histologically classified as grade 2 (molGBMA2) was compared with IDH-mutant astrocytoma grade 2 (IDH-mut A2), and molGBM histologically classified as grade 3 (molGBM A3) was compared with IDH-mutant astrocytoma grade 3 (IDH-mut A3). This stratified comparison was performed to determine whether molGBM follows the clinical course of IDH-mutant astrocytoma with similar histological morphology or instead behaves more similarly to glioblastoma.

The study was conducted in accordance with the principles of the Declaration of Helsinki. At the authors’ institution, no formal ethical approval is required for retrospective analyses of anonymized clinical data.

### Neuropathological analyses

All tumors examined were classified and graded according to the criteria of the 5th edition of the WHO Classification [1]. Molecular testing was performed as part of routine integrated neuropathological diagnostics and was not identical in all cases, reflecting the retrospective study period and evolving diagnostic standards. The expression status of Ki-67, IDH1 R132H, p53, ATRX, GFAP, and MAP2 was determined immunohistochemically in all cases. If the tumor was midline-related, the expression status of H3 K27M and H3 K27me3 was additionally assessed by immunohistochemistry. If the patient was younger than 30 years of age or exhibited a negative IDH status in combination with a loss of nuclear expression of ATRX, expression of H3 G34R was tested immunohistochemically. In the case of IDH1 R132H negativity, patients aged 55 years or younger subsequently underwent sequencing of TERT, IDH1, IDH2, H3F3A, and TP53 using a laboratory-internal validated NGS panel. If all three markers exhibited a wild-type status, the tumor tissue was examined for EGFR amplification using FISH analysis. Following cIMPACT-NOW Update 3, which recommended molecular criteria for classifying IDH-wildtype diffuse astrocytic gliomas as glioblastoma [2], histologically lower-grade IDH-wildtype diffuse astrocytic gliomas were assessed for glioblastoma-defining molecular alterations, including TERT promoter mutation and EGFR amplification, irrespective of patient age. MGMT status was determined by pyrosequencing only for a part of the hisGBM or molGBM tumors. Chromosome + 7/−10 copy-number status was not routinely assessed in this retrospective cohort and was therefore not used as a systematic diagnostic criterion for molGBM classification.

### Data analysis

Continuous variables are reported as median and range and were compared between groups using the Wilcoxon rank-sum test. Categorical variables are reported as counts and percentages and were compared using Fisher’s exact test. For the primary comparison, patients with molGBM were compared with patients with hisGBM.

OS was defined as the time from initial surgery to death. PFS was defined as the time from initial surgery to radiological progression, or death, whichever occurred first. Progression was determined retrospectively from medical records, routine follow-up MRI reports, and treating physicians’ documentation. Formal retrospective reassessment according to RANO criteria and central neuroradiological review were not performed; therefore, PFS analyses were considered exploratory. OS and PFS were estimated using the Kaplan-Meier method and are reported as median survival with 95% confidence intervals (CI). Survival differences were assessed using the log-rank test. For the primary survival analysis, OS and PFS were compared between molGBM and hisGBM. To further assess the association between tumor group and OS, Cox proportional hazards regression was performed. First, a univariable Cox model including tumor group only was calculated. Subsequently, a multivariable Cox model was fitted including tumor group, age, and KPS. The proportional hazards assumption was assessed using Schoenfeld residuals. Furthermore, exploratory subgroup analyses were performed separately in biopsy-only and resected patients. In addition, an exploratory multivariable Cox model was fitted including tumor group, age, KPS, and surgical strategy, categorized as biopsy versus resection. KPS was entered as a continuous variable per 10-point increase.

Within the molGBM cohort, an exploratory analysis was performed according to the diagnostic molecular alteration. Tumors with TERT promoter mutation without EGFR amplification were compared with tumors showing EGFR amplification, irrespective of TERT status. In addition, an exploratory analysis was performed after exclusion of molGBM cases defined by TERT promoter mutation without EGFR amplification. In this analysis, the remaining EGFR-amplified molGBM cases were compared with the hisGBM control cohort. Univariable Cox regression was used to estimate hazard ratios.

As a sensitivity analysis, propensity score matching was performed to account for baseline clinical differences between molGBM and hisGBM. Propensity scores were estimated using logistic regression including age, KPS, and sex. Patients were matched using nearest-neighbor matching without replacement in a 1:1. Covariate balance before and after matching was assessed using standardized mean differences. OS and PFS were then compared between matched groups using Kaplan-Meier analysis and the log-rank test. Cox regression with robust standard errors accounting for matched subclasses was additionally performed in the matched cohort. As an exploratory analysis, an additional extended propensity score model was fitted including age, sex, KPS, surgical strategy, contrast enhancement, seizure presentation, and focal neurological deficit. Surgical strategy was categorized as biopsy versus resection, and focal neurological deficit was defined as the presence of motor deficit, aphasia, visual disturbance, cognitive symptoms, or gait disturbance. Patients were matched 1:1 using nearest-neighbor matching without replacement and a caliper of 0.2 standard deviations of the logit of the propensity score.

The additional survival analyses of molGBM relative to IDH-mutant astrocytoma of corresponding histological grade were performed using Kaplan-Meier estimates and log-rank tests.

All analyses and plots were performed in R Studio [Version 2025.09.2 + 418 (2025.09.2 + 418); Copyright (C) 2025 by Posit Software, PBC]. Statistical significance was defined as *p* < 0.05.

## Results

### Cohort description

A total of 438 patients diagnosed with CNS WHO grade 2 to 4 gliomas between 2017 and 2024 were retrospectively reviewed. Among these, a total of 34 patients were identified as having IDH-wildtype astrocytomas that were histologically graded as CNS WHO grade 2 or 3 but harbored either a TERT promoter mutation or EGFR amplification. Based on the 2021 WHO classification criteria [1], these tumors were reclassified as molGBM due to their defining molecular alterations despite lower histological grade. For each molGBM case, approximately three patients with hisGBM diagnosed closest in time were selected as controls, resulting in a total of 103 control cases.

Patients diagnosed with molGBM were significantly younger than patients with hisGBM, with a median age of 61 years (range 38–77) compared with 68 years (range 30–89; *p* < 0.001). Patients with molGBM also presented with a higher KPS at admission than patients with hisGBM, with a median KPS of 90 (range 60–90) versus 80 (range 50–90; *p* < 0.001). Both groups showed a predominance of male patients, with no significant difference in sex distribution between molGBM and hisGBM (male: 65% vs. 59%; *p* = 0.7). No statistically significant differences were observed regarding arterial hypertension (47% vs. 29%; *p* = 0.062), diabetes mellitus (8.8% vs. 3.9%; *p* = 0.4), or coronary artery disease (8.8% vs. 3.9%; *p* = 0.4). Patients’ baseline characteristics are summarized in Table 1.

**Table 1:** Baseline characteristics and treatment regimens

Characteristic	molGBM *N* = 34^*1*^	hisGBM *N* = 103^*1*^	*p*-value^2^
**Age**,** years**	61 (38–77)	68 (30–89)	< 0.001
**Sex**			0.7
F	12 (35%)	42 (41%)	
M	22 (65%)	61 (59%)	
**Karnofsky Performance Status**	90 (60–90)	80 (50–90)	< 0.001
**Contrast enhancement**	8 (24%)	100 (97%)	< 0.001
**Surgical procedure**			< 0.001
Biopsy	23 (68%)	4 (3.9%)	
Subtotal resection	8 (24%)	46 (45%)	
Gross total resection	3 (8.8%)	53 (51%)	
**Adjuvant therapy**			0.4
Chemoradiotherapy	27 (79%)	73 (71%)	
None	3 (8.8%)	7 (6.8%)	
Radiation	4 (12%)	23 (22%)	
**MGMT promoter methylation**			> 0.9
negative	16 (64%)	46 (65%)	
positive	9 (36%)	25 (35%)	
Unknown	9	32	
**Ki-67 index**,** %**	7 (2–70)	30 (3–90)	< 0.001
Unknown	6	11	
**Comorbidities**			
Arterial hypertension	16 (47%)	30 (29%)	0.062
Diabetes mellitus	3 (8.8%)	4 (3.9%)	0.4
Coronary artery disease	3 (8.8%)	4 (3.9%)	0.4
**Symptoms**			
Seizure	19 (56%)	29 (28%)	0.006
Motor deficit	1 (2.9%)	25 (24%)	0.005
Aphasia	2 (5.9%)	32 (31%)	0.003
Cognitive/behavioral impairment	5 (15%)	26 (25%)	0.2
Visual deficit	1 (2.9%)	15 (15%)	0.12
Gait disorder	0 (0%)	13 (13%)	0.038

^*1*^Median (Min–Max); n (%)

^*2*^Wilcoxon rank sum test; Fisher’s exact test

The secondary control groups included 17 patients with IDH-mut A2, and 14 patients with IDH-mut A3. When comparing patients with molGBM A2, to those with IDH-mut A2, no significant differences were observed in sex distribution (*p* = 0.29), KPS (*p* > 0.99), or the presence of diabetes mellitus (*p* = 0.08). However, patients in the molGBM A2 group were significantly older (*p* < 0.0001) and had a higher prevalence of arterial hypertension (*p* = 0.0004). In contrast, comparison between molGBM A3 and IDH-mut A3 revealed no significant differences with respect to age (*p* = 0.09), sex distribution (*p* = 0.06), KPS (*p* = 0.21), arterial hypertension (*p* = 0.23), or diabetes mellitus (*p* > 0.99).

### Clinical presentation and imaging findings

The majority of patients with molGBM presented with seizures (56%). Further presenting symptoms included cognitive or behavioral impairment (15%), aphasia (6%), motor deficits (3%), and visual deficits (3%). In patients with hisGBM, the most common symptoms were aphasia (31%), seizures (28%), cognitive or behavioural impairment (25%), and motor deficits (24%), followed by visual deficits (15%) and gait disorders (13%).

**Fig. 2:**
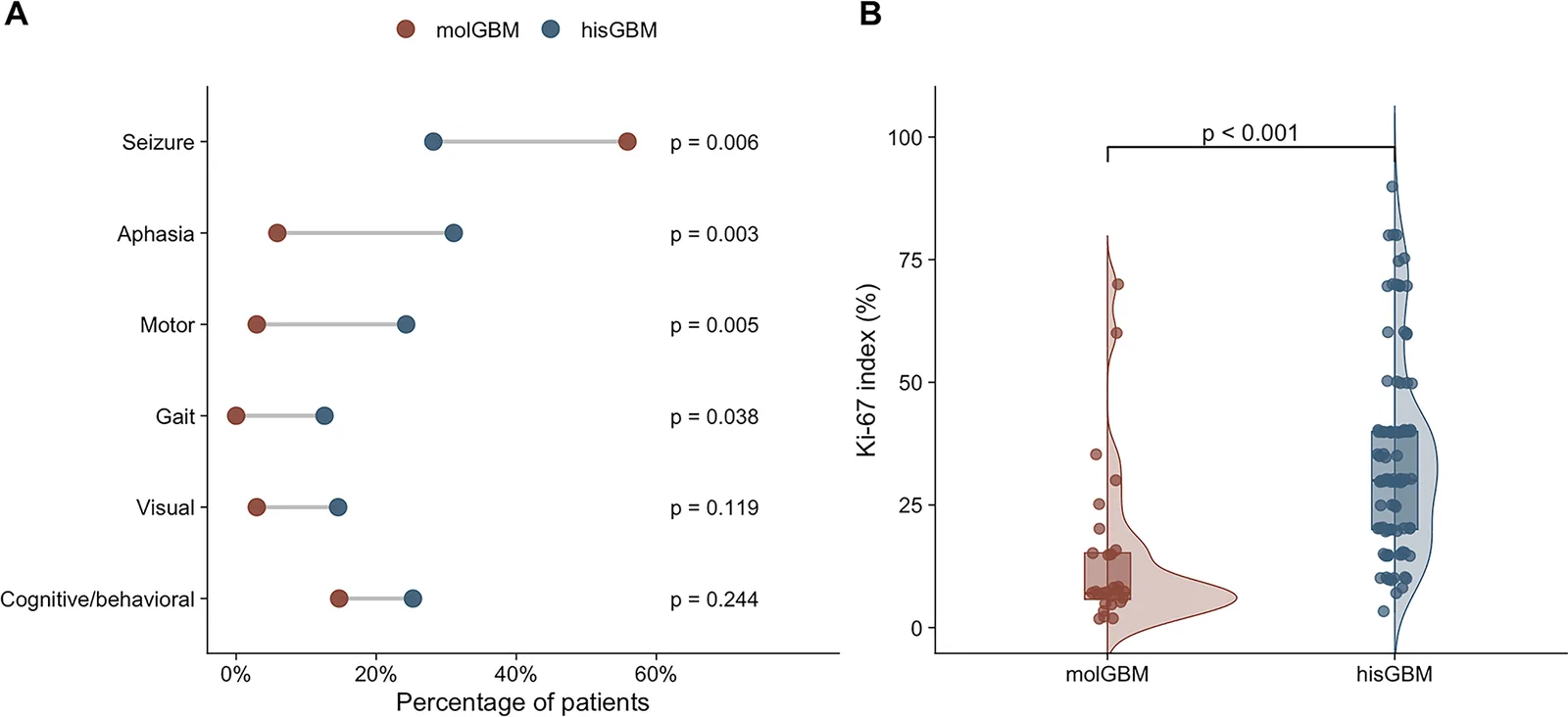
Clinical presentation and proliferative activity in molGBM and hisGBM. (**A**) Dumbbell plot showing the frequency of presenting symptoms in patients with molGBM and hisGBM. Dots indicate the proportion of patients presenting with each symptom, and connecting lines visualize the absolute between-group difference. Symptoms are ordered according to the magnitude of the difference between groups. (**B**) Ki-67 proliferation index in molGBM and hisGBM. Half-violin plots with boxplots and individual data points show the distribution of Ki-67 indices. Values were available for 28/34 molGBM and 92/103 hisGBM cases. Missing values were not plotted and were excluded from the statistical comparison; no imputation was performed

Group comparison showed that seizures were significantly more frequent in molGBM than in hisGBM (56% vs. 28%; *p* = 0.006). In contrast, neurological deficits were more common in hisGBM, including motor deficits (24% vs. 2.9%; *p* = 0.005), aphasia (31% vs. 5.9%; *p* = 0.003), and gait disorders (13% vs. 0%; *p* = 0.038). Cognitive or behavioural impairment and visual deficits did not differ significantly between groups (see Fig. 2A).

Contrast enhancement was present in 24% of molGBM and 97% of hisGBM cases (*p* < 0.001), indicating that the majority of molGBM presented as non-enhancing or only minimally enhancing lesions, whereas almost all hisGBM showed contrast enhancement.

### Neuropathological findings

**Fig. 3:**
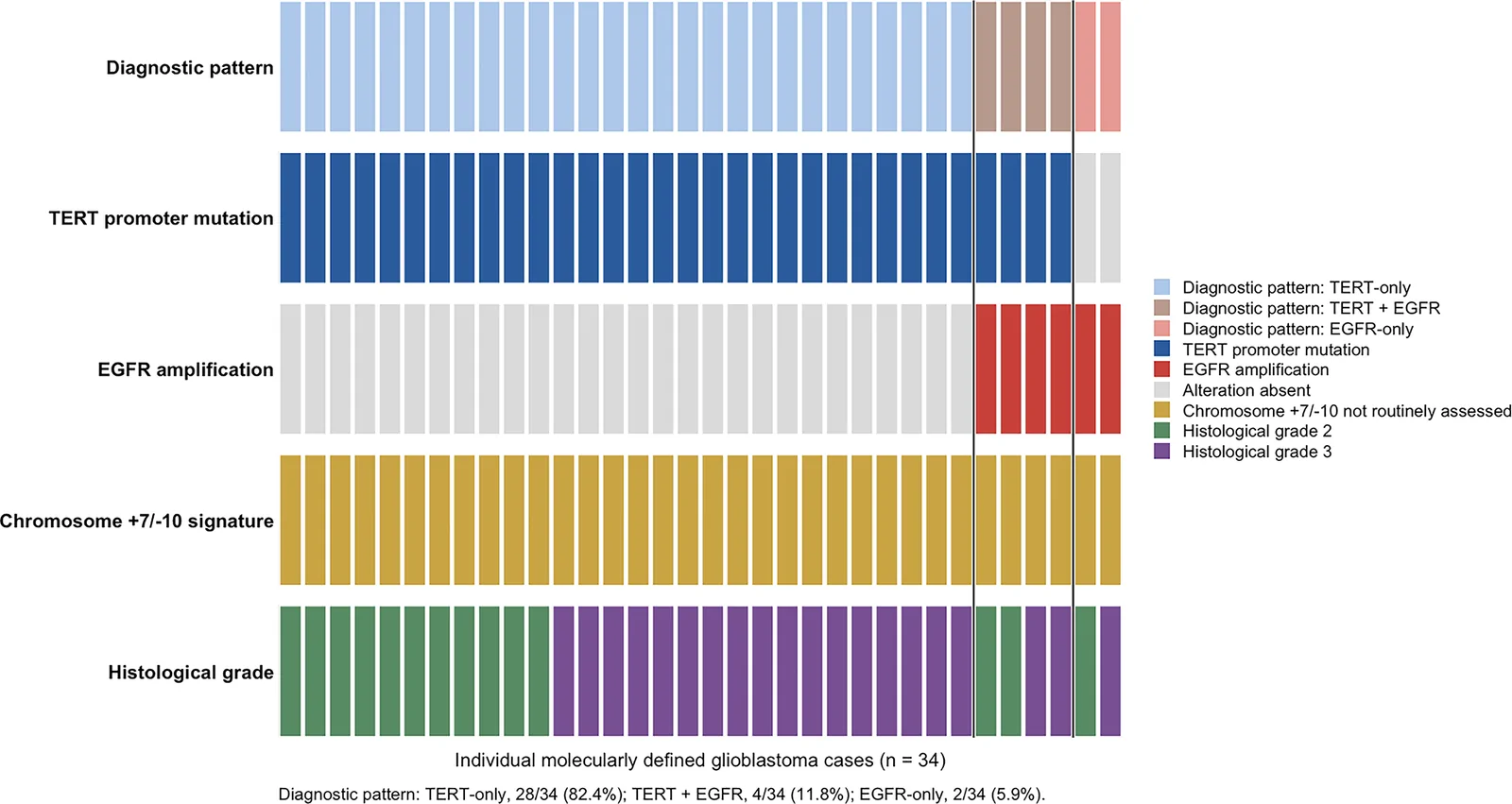
Molecular diagnostic patterns in molecularly defined glioblastoma. Each column represents one individual molGBM case (*n* = 34). Rows show the molecular diagnostic pattern, TERT promoter mutation status, EGFR amplification status, chromosome + 7/−10 assessment, and histological grade at initial diagnosis. Diagnostic patterns were categorized as TERT promoter mutation without EGFR amplification (TERT-only, *n* = 28, 82.4%), combined TERT promoter mutation and EGFR amplification (TERT + EGFR, *n* = 4, 11.8%), or EGFR amplification without TERT promoter mutation (EGFR-only, *n* = 2, 5.9%). Chromosome + 7/−10 status was not routinely assessed. Histological grade refers to the initial histological classification as grade 2 (*n* = 14) or grade 3 (*n* = 20)

**Fig. 4:**
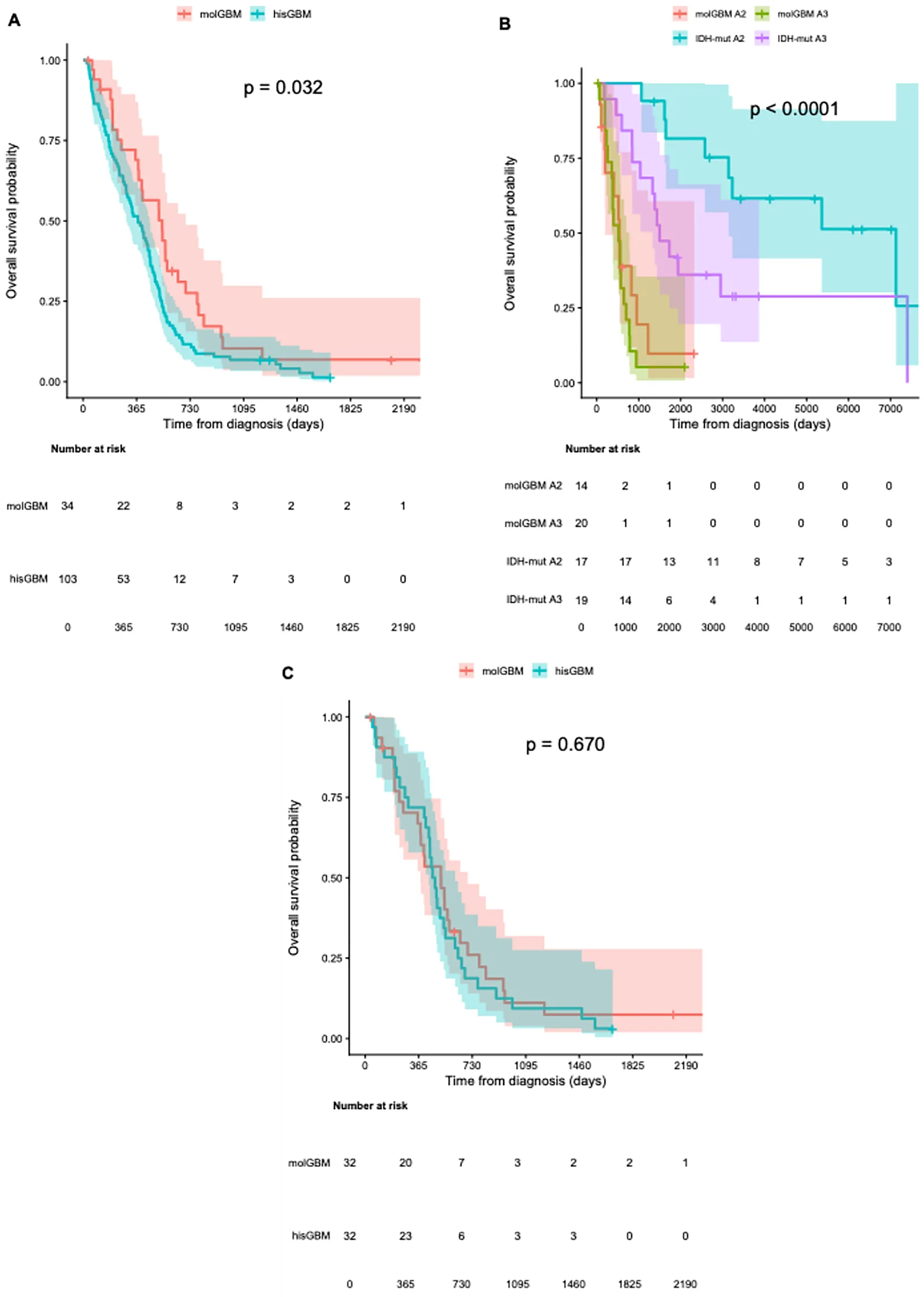
Overall survival in molGBM, hisGBM, IDH-mutant astrocytoma, and the propensity score-matched cohort. (**A**) Kaplan-Meier analysis of overall survival in patients with molGBM and hisGBM. Patients with molGBM showed significantly longer overall survival compared with patients with hisGBM in the unmatched cohort. (**B**) Kaplan-Meier analysis of overall survival stratified by histological grade and molecular classification. Patients with molGBM histologically classified as grade 2 or grade 3 are compared with patients with IDH-mutant astrocytoma of corresponding histological grade. (**C**) Kaplan-Meier analysis of overall survival in the propensity score-matched cohort of molGBM and hisGBM patients. Shaded areas indicate 95% confidence intervals. Survival differences were assessed using the log-rank test

All tumors classified as molGBM were IDH-wildtype astrocytomas lacking necrosis and vascular proliferation. A total of 14 patients with molGBM were histologically graded as molGBM A2 while 20 were graded as molGBM A3, applying the WHO criteria for IDH-mutated diffuse astrocytomas. Among these 34 molGBM cases, 32 tumors (94.1%) harbored a TERT promoter mutation and 6 tumors (17.6%) showed EGFR amplification. Overall, 28 tumors (82.4%) harbored a TERT promoter mutation without EGFR amplification, 4 tumors (11.8%) showed both TERT promoter mutation and EGFR amplification, and 2 tumors (5.9%) showed EGFR amplification without TERT promoter mutation. The molecular diagnostic patterns of all molGBM cases are summarized in Fig. 3. Chromosome + 7/−10 copy-number status was not routinely assessed. TERT and EGFR status were not consistently analyzed in hisGBM. MGMT promoter methylation status was available in 25 of 34 patients with molGBM (74%) and in 71 of 103 patients with hisGBM (69%). Among patients with available MGMT status, promoter methylation was present in 36% of molGBM and 35% of hisGBM cases, with no significant difference between groups (*p* > 0.9). Ki-67 index was available in 28 of 34 patients with molGBM (82%) and in 92 of 103 patients with hisGBM (89%), and was significantly lower in molGBM than in hisGBM, with a median of 7% (range 2–70) versus 30% (range 3–90), respectively (*p* < 0.001; Fig. 2B). In 4 of 6 resected recurrent molGBM, vascular proliferation was observed, whereas it had not been present in the corresponding primary tumors (Fig. 4).

### Surgical management and adjuvant therapy

Surgery was performed according to departmental standards [9, 10]. The majority of patients with molGBM underwent diagnostic biopsy as the primary surgical intervention (68%), whereas most patients with hisGBM underwent tumor resection (96%), reflecting a significant difference in surgical management between groups (*p* < 0.001). Within the molGBM cohort, subtotal resection was performed in 24% of patients and gross-total resection in 9%. In contrast, subtotal and gross-total resections were performed in 45% and 51% of patients with hisGBM, respectively. Diagnostic biopsy was performed in only 4% of hisGBM cases.

Adjuvant treatment did not differ significantly between groups (*p* = 0.4). In the molGBM group, 79% of patients received chemoradiotherapy, 12% received radiotherapy alone, and 9% received no adjuvant treatment. In the hisGBM group, 71% received chemoradiotherapy, 22% received radiotherapy alone, and 7% received no adjuvant treatment.

### Overall survival and progression-free survival

Median follow-up by reverse Kaplan-Meier estimation was 1688 days overall, 2104 days in molGBM, and 1688 days in hisGBM. OS was significantly longer in the molGBM group compared with the hisGBM group, with a median OS of 539 days (95% CI 379–699) versus 374 days (95% CI 296–452), respectively (log-rank *p* = 0.032). PFS was also significantly longer in molGBM than in hisGBM, with a median PFS of 340 days (95% CI 188–541) versus 184 days (95% CI 149–281), respectively (log-rank *p* = 0.015). Survival analyses are summarized in Table 2.

**Table 2:** Survival outcomes in molGBM and hisGBM

Endpoint	Group	Events, *n*/*N* (%)	Median survival, days (95% CI)	Log-rank *p*-value
Overall survival	molGBM	29/34 (85.3%)	539 (379–699)	0.032
	hisGBM	100/103 (97.1%)	374 (296–452)	
Progression-free survival	molGBM	30/34 (88.2%)	340 (188–541)	0.015
	hisGBM	102/103 (99%)	184 (149–281)	

In univariable Cox regression, hisGBM was associated with a significantly higher risk of death compared with molGBM (HR 1.57, 95% CI 1.04–2.38; *p* = 0.033). After adjustment for age and KPS, this association was attenuated and no longer statistically significant (HR 1.21, 95% CI 0.77–1.90; *p* = 0.411). In the multivariable model, higher KPS remained significantly associated with improved OS (HR 0.76 per 10-point increase, 95% CI 0.63–0.92; *p* = 0.005), whereas age was not significantly associated with OS (HR 1.01 per year, 95% CI 1.00–1.03; *p* = 0.109). The proportional hazards assumption was not violated (global *p* = 0.55).

In the biopsy-only subgroup, 23 patients had molGBM and 4 had hisGBM. Median OS was comparable between groups (517 days in molGBM vs. 540.5 days in hisGBM; log-rank *p* = 0.844; HR 1.12, 95% CI 0.37–3.36; *p* = 0.844). In the resection subgroup, 11 patients had molGBM and 99 had hisGBM. MolGBM showed a non significant trend toward longer OS compared with hisGBM (median OS 550.5 vs. 342 days; log-rank *p* = 0.299; HR 1.41, 95% CI 0.73–2.72; *p* = 0.301). In the exploratory multivariable Cox model including tumor group, age, KPS, and surgical strategy, tumor group was not associated with OS (HR 1.05, 95% CI 0.59–1.85; *p* = 0.878). Age was not significantly associated with OS (HR 1.01 per year, 95% CI 1.00–1.03; *p* = 0.091), whereas higher KPS remained significantly associated with longer OS (HR 0.77 per 10-point increase, 95% CI 0.64–0.93; *p* = 0.008). Surgical strategy was also not significantly associated with OS in this exploratory model (HR 0.79, 95% CI 0.43–1.44; *p* = 0.437).

Within the molGBM cohort, 28 tumors harbored a TERT promoter mutation without EGFR amplification, whereas 6 tumors showed EGFR amplification. Median OS was 539 days in the TERT-only subgroup and 552 days in the EGFR-amplified subgroup. No significant OS difference was observed between groups (HR 0.88, 95% CI 0.35–2.17; *p* = 0.773). After exclusion of the 28 TERT-only tumors, the remaining 6 EGFR-amplified molGBM cases showed descriptively longer OS than selected hisGBM controls, although this difference was not statistically significant (median OS 552 vs. 374 days; HR 1.53, 95% CI 0.67–3.51; *p* = 0.313).

No significant differences in OS or PFS were observed between patients with molGBM A2 and molGBM A3. Median OS was 539 days in molGBM A2 (95% CI 201–not reached) and 516 days in molGBM A3 (95% CI 376–778; log-rank *p* = 0.409). Median PFS was 301 days in molGBM A2 (95% CI 187–not reached) and 340 days in molGBM A3 (95% CI 188–561; log-rank *p* = 0.369).

In contrast, molGBM showed significantly shorter survival than IDH-mutant astrocytoma of corresponding histological grade. Among histological grade 2 tumours, median OS was 539 days in molGBM A2 (95% CI 201–not reached) compared with 7,129 days in IDH-mut A2 (95% CI 3,140–not reached; log-rank *p* < 0.001). Median PFS was 301 days in molGBM A2 (95% CI 187–not reached) compared with 1,330 days in IDH-mut A2 (95% CI 550–4,080; log-rank *p* = 0.006). Among histological grade 3 tumours, median OS was 516 days in molGBM A3 (95% CI 376–778) compared with 1,498 days in IDH-mut A3 (95% CI 1,330–not reached; log-rank *p* < 0.001). Median PFS was likewise shorter in molGBM A3 than in IDH-mut A3, with a median PFS of 340 days versus 733 days, respectively (log-rank *p* = 0.005). Kaplan-Meier curves for OS are shown in Fig. 2.

### Propensity score-matched sensitivity analysis of survival outcomes

Matching substantially improved baseline covariate balance, with standardized mean differences for age, KPS, and sex decreasing from 0.75, 0.94, and 0.11 before matching to 0.15, 0.04, and 0.07 after matching, respectively. However, surgical strategy remained markedly imbalanced. Before matching, biopsy was performed in 67.6% of molGBM and 3.9% of hisGBM cases (SMD = 1.78); after matching, biopsy rates were 71.9% and 6.3%, respectively (SMD = 1.82). In this matched cohort, OS no longer differed significantly between groups, with a median OS of 517 days in molGBM (95% CI 376–699) compared with 468 days in hisGBM (95% CI 434–613; log-rank *p* = 0.670). Cox regression with robust standard errors accounting for matched subclasses showed no significant association between tumor group and OS (HR 0.89, 95% CI 0.55–1.46; *p* = 0.655). Similarly, no significant difference in PFS was observed in the matched cohort. Median PFS was 357 days in molGBM (95% CI 187–549) and 413 days in hisGBM (95% CI 271–490), with no significant difference by log-rank testing (*p* = 0.269). Robust Cox regression confirmed the absence of a significant association between tumor group and PFS (HR 0.74, 95% CI 0.45–1.20; *p* = 0.224). These results are summarized in Table 3.

**Table 3:** Survival outcomes in the propensity score-matched cohort

Endpoint	Group	Events, *n*/*N* (%)	Median survival, days (95% CI)	Log-rank *p*-value
Overall survival	hisGBM	31/32 (96.9%)	468 (434–613)	0.670
	molGBM	27/32 (84.4%)	517 (376–699)	
Progression-free survival	hisGBM	32/32 (100%)	413 (271–490)	0.269
	molGBM	28/32 (87.5%)	357 (187–549)	

The extended caliper-matched propensity score sensitivity analysis yielded only 7 matched pairs, indicating limited covariate overlap between molGBM and hisGBM when surgical strategy and clinical-radiological variables were included. Balance improved for several variables, including KPS (SMD 0.774 before matching vs. 0 after matching), surgical strategy (SMD 1.782 vs. 0), and contrast enhancement (SMD 2.280 vs. 0). Focal neurological deficit was also balanced after matching (SMD 0), whereas residual imbalance remained for age (SMD 0.295), sex (SMD 0.354), and seizure presentation (SMD 0.603). Median OS was 541 days in molGBM and 490 days in hisGBM, with no significant difference between groups (HR 1.32, 95% CI 0.40–4.39; *p* = 0.651).

## Discussion

In this retrospective single-center study, we evaluated the clinical characteristics and outcomes of patients with molecularly defined glioblastoma according to the 2021 WHO classification [1]. Compared with patients with histologically defined glioblastoma, individuals with molGBM were younger, had a higher functional status at admission, and more frequently presented with seizures, whereas hisGBM more commonly presented with focal neurological deficits such as aphasia, motor deficits, and gait disturbance. Radiologically, molGBM showed contrast enhancement far less frequently. Neuropathologically, molGBM displayed a markedly lower Ki-67 index, while MGMT promoter methylation did not differ between groups. Surgical management differed substantially, with biopsy being the predominant procedure in molGBM and resection being performed in nearly all patients with hisGBM. In unadjusted survival analyses, molGBM was associated with significantly longer OS and PFS than hisGBM; however, this survival advantage was attenuated after adjustment for age and KPS and was no longer evident after propensity score matching. Within the molGBM cohort, histological grade 2 and grade 3 tumors did not differ significantly in OS or PFS. In contrast, outcomes remained clearly inferior to those of IDH-mutant astrocytomas of corresponding histological grade.

Following the cIMPACT-NOW recommendations and their incorporation into the 2021 WHO classification of CNS tumors, several studies have compared molecularly defined and histologically defined glioblastoma, reporting heterogeneous results regarding survival outcomes. Several cohorts found similar OS between both groups, supporting the WHO 2021 concept that IDH-wildtype diffuse astrocytic gliomas with molecular GBM features behave within the glioblastoma spectrum [11–14]. Other studies reported longer OS in molGBM [15–18]. A possible reason for these divergent findings may be differences in baseline characteristics, notably, younger age and better preoperative performance scores among patients with molGBM compared to hisGBM, as observed both in our cohort and in previous studies [15–17]. However, adjustment for baseline factors does not fully resolve the issue, because surgical management differs between molGBM and hisGBM. In our cohort, molGBM was predominantly biopsied, whereas hisGBM was mostly resected, which aligns with previous cohorts [13, 16, 18, 19]. This likely reflects the preoperative appearance of many molGBM cases as presumed lower-grade diffuse gliomas, with limited contrast enhancement and diffuse infiltrative growth. Therefore, biopsy-only in these cases reflected individualized surgical risk-benefit assessment and the need to obtain tissue for integrated molecular diagnosis, rather than a general biopsy-only policy. This variable is difficult to adjust for or include in propensity matching because surgical strategy is not a neutral confounder. Rather, it reflects tumor phenotype, contrast enhancement, anatomical location, eloquence and perceived resectability, all of which may themselves affect outcome. Therefore, adjusting or matching for surgical strategy may introduce additional bias by conditioning on a variable that is closely linked to the preoperative imaging phenotype and clinical presentation, particularly because biopsy may reflect different clinical scenarios in molGBM and hisGBM: in hisGBM, biopsy more often indicated unresectability or unfavorable clinical status, whereas in molGBM it often reflected a presumed lower-grade, diffuse infiltrative growth, or minimally enhancing lesion requiring tissue for integrated diagnosis. Robinson et al. showed that survival interpretation changes when OS is analyzed by surgical extent, with OS differences after resection but not after biopsy [18]. Therefore, the absence of an independent OS advantage after adjustment or matching in our study should not be interpreted as evidence that molGBM and hisGBM are identical. Rather, the loss of significance after adjustment or matching indicates that the unadjusted OS advantage should not be interpreted as evidence of an independent survival benefit of molGBM, but as reflecting the influence of baseline clinical context. Given the relatively small molGBM cohort and the width of the confidence intervals, these findings should not be interpreted as demonstrating equivalence between molGBM and hisGBM, as clinically meaningful survival differences cannot be excluded. Stratification by surgical strategy did not reveal statistically significant OS differences within the biopsy-only or resection subgroups. However, these analyses were exploratory and underpowered, particularly because of the very small number of biopsy-only hisGBM cases and resected molGBM cases. Notably, within the resection subgroup, molGBM showed descriptively longer OS than hisGBM (median OS 551 vs. 342 days), arguing against the observed survival pattern being explained primarily by surgical strategy alone. At the same time, the extended caliper-matched analysis, which included surgical strategy and clinical-radiological presentation, retained only 7 matched pairs, confirming limited covariate overlap in this cohort. Thus, these analyses do not eliminate surgical confounding, but suggest that survival comparisons between molGBM and hisGBM were strongly influenced by differences in baseline clinical-radiological presentation and treatment selection, which could not be fully resolved by statistical adjustment in our cohort.

In our series, no significant survival differences were observed between molGBM A2 and A3, a finding that agrees with reports by Wijnenga et al. and Keric et al. [20, 21], but contrasts with results from Berzero et al. and Nakasu et al. [22, 23], who reported worse outcomes in higher histological grades. Consistent with previous reports, IDH-mutant astrocytomas of corresponding histological grade showed longer survival than molGBM [24]. However, due to small group sizes and lack of multivariable adjustment, these comparisons were intended as exploratory prognostic context rather than conclusive evidence.

Our data indicate that molGBM tends to present more frequently as non-enhancing lesions and is associated with a less severe clinical presentation and younger age. These observations are in line with several prior reports describing younger age [15, 25] and better preoperative KPS [15, 16, 26] among patients with molGBM compared to hisGBM. Other studies demonstrated a higher incidence of seizures and fewer neurologic deficits in molGBM, supporting the notion of a less debilitating initial presentation [16–18, 25]. The predominance of non-enhancing tumors and the markedly lower Ki-67 index observed in our cohort are also consistent with previously published series [15, 16, 25]. Both, a low proliferative index and absence of contrast enhancement have been linked to improved survival outcomes in glioblastoma [13, 27–29]. In the present cohort, these features support the presence of a distinct clinical-radiological and proliferative phenotype, but do not establish molGBM as an independently more favorable biological entity. Taken together, our data suggest that molGBM is characterized by a distinct clinical-radiological and proliferative profile; however, the partially conflicting evidence in the literature highlights the need for larger studies to determine whether this reflects biological heterogeneity.

Of particular interest, 2/3 of resected recurrent molGBM in our cohort demonstrated histological progression to conventional GBM with the development of microvascular proliferation, which was absent in the corresponding primary tumors. This observation is consistent with findings by Ramos-Fresnedo and colleagues, who hypothesized that molGBM may represent conventional GBM at an earlier stage [30]. However, given the small number of resected recurrent tumors and the inherent risk of selection and sampling bias, this finding should be interpreted as hypothesis-generating only. While this model is plausible, the repeatedly reported distinct clinical presentation argues against a simple chronological continuum. Future studies incorporating longitudinal molecular profiling will be essential to elucidate the genetic events driving this histological transformation and to refine the biological definition of molGBM.

More recently, cIMPACT-NOW update 11 recommended greater caution when diagnosing histologically lower-grade, IDH-wildtype diffuse astrocytic tumors as glioblastoma based on an isolated TERT promoter mutation [31]. Instead, clinical, radiological, histological, and additional molecular findings, ideally including DNA methylation profiling, should be integrated [31]. This is directly relevant to our cohort, in which 28 of 34 molGBM appeared to be classified by TERT promoter mutation alone. However, the exploratory molecular subgroup analyses argue against the observed survival pattern being driven solely by TERT-only tumors. Survival within molGBM did not differ between TERT-only and EGFR-amplified tumors. In addition, after exclusion of TERT-only cases, the remaining EGFR-amplified molGBM cases still showed descriptively longer OS than hisGBM, although this difference was not statistically significant (median OS 552 vs. 374 days). However, this analysis was strongly limited by the small number of EGFR-amplified molGBM cases (*n* = 6) and the absence of DNA methylation profiling. Therefore, these findings should be interpreted as descriptive only and cannot exclude molecular heterogeneity or inadvertent inclusion of tumors with more favorable biology. The distinct clinical-radiological phenotype observed in these patients may therefore reflect heterogeneity within tumors currently classified as molGBM. However, without DNA methylation profiling and broader molecular characterization, conclusions regarding distinct molecular subgroups remain speculative. At the same time, histological progression at recurrence and inferior survival compared with IDH-mutant astrocytoma support malignant potential in at least a subset. Methylation profiling would help determine how many tumors remain aligned with the glioblastoma, IDH-wildtype methylation family under the proposed criteria. Importantly, cIMPACT-NOW update 11 provides interim diagnostic guidance rather than a formal revision of WHO CNS5. Thus, the precise definition and classification of these tumors remain unsettled pending the next WHO classification of CNS tumors. Until then, transparent reporting of the histological and molecular basis of diagnosis is essential, particularly for tumors defined by an isolated TERT promoter mutation.

This study has several limitations. Its retrospective single-center design carries a risk of selection bias. Biopsy-only diagnosis represents an important limitation, as the absence of necrosis or microvascular proliferation in sampled tissue does not exclude these features in unsampled tumor regions. Although integrated molecular diagnostics supported classification as molGBM by demonstrating glioblastoma-defining molecular alterations, histological undersampling may still have contributed to classification as molGBM rather than hisGBM in a subset of cases. DNA methylation profiling was not available, limiting confirmation that all tumors aligned with the glioblastoma, IDH-wildtype methylation family and limiting exclusion of other methylation-defined entities. Chromosome + 7/−10 copy-number status was not routinely available, and the molGBM cohort was predominantly composed of tumors with TERT promoter mutation without EGFR amplification; therefore, our findings should not be interpreted as equally representative of all molGBM-defining alterations. H3F3A status was not assessed systematically; although ATRX immunohistochemistry made diffuse hemispheric glioma, H3 G34-mutant unlikely in most cases, this entity could not be excluded with certainty; and none of the tumors arose in a midline location, making diffuse midline glioma unlikely. In addition, systematic broad NGS characterization was not available. Furthermore, the high proportion of biopsy-only procedures in molGBM represents an additional limitation, as it limits assessment of resection-related effects and contributes to treatment-related confounding in comparisons with hisGBM. Also, no systematic central neuroradiological review, including standardized volumetric tumor assessment or formal RANO-based reassessment of progression, was performed. Finally, molecular data were incomplete in a subset of patients, particularly for MGMT promoter methylation, which may have introduced residual confounding and precluded inclusion of this variable in selected multivariable and propensity score analyses.

## Conclusions

In conclusion, molGBM in our cohort showed distinct clinical-radiological and proliferative characteristics within the IDH-wildtype glioblastoma spectrum. Although OS was longer than in hisGBM in unadjusted analyses, this survival advantage was attenuated after adjustment and propensity score matching, indicating substantial influence of baseline clinical-radiological characteristics rather than an established independent prognostic effect. Larger cohorts with DNA methylation profiling and systematic + 7/−10 assessment are needed to determine whether these clinical-radiological differences reflect underlying biological heterogeneity.

## Data Availability

The datasets generated and analyzed during the current study are available from the corresponding author on reasonable request.
